# Trials special series - the collection, analysis and reporting of adverse events in randomised controlled trials

**DOI:** 10.1186/s13063-025-08861-3

**Published:** 2025-05-12

**Authors:** Rachel Phillips, Victoria Cornelius

**Affiliations:** Imperial Clinical Trials Unit, School of Public Health, Imperial College London, Stadium House, 68 Wood Lane, London, W12 7RH UK

## Introduction

### Background

While the design and analysis of randomised controlled trials has progressed rapidly in the last decade, the analysis of harm outcomes has seen minimal change [1–3]. Integral to assessing harm is what and how data is collected. Considerations of all these aspects vary across the different types of interventions e.g. pharmaceutical, surgical, behavioural etc. While there has been an increase in research to progress how data should be collected, synthesised and reported for harm outcomes, there is little evidence of progress in implementing these approaches and the analysis and reporting of harm outcomes remains rudimentary [3].

In this special series, Trials invited submissions that related to the collection, analysis and reporting of adverse events in clinical trials. To date twelve articles have been published in the series. In this editorial, we summarise each of these submissions and briefly discuss the implications on applied clinical trials and future methodological research.

## Overview of submissions

The articles in this series cover the areas of trial conduct, analysis, reporting, and public involvement.

- Trial conduct involves all the steps taken to set-up and deliver a trial.
- Trial analysis refers to the (statistical) steps taken to summarise and allow inference to be drawn from collected data.
- Trial reporting describes the process of presenting and communicating trial results.
- Public involvement describes the activities carried out with or by members of the public which influences and shapes a trial and/or trial processes [4].

### Trial conduct

Guidance and standards for the assessments of harm have been driven by the need to regulate trials of pharmacological interventions. Historically *the collection and reporting of adverse events in trials of complex interventions* such as behavioural interventions have received less attention, largely due to differences in the intervention adoption process. Researchers are now seeking to redress this gap, for example the Recording HArms in Behavioural change Intervention Trials (RHABIT) initiative developed guidance to improve the recording of harms in trials of behavioural change interventions [5]. Two articles in this special series look to build on this research. Frantz et. al. use a case study to demonstrate the development and testing of a tool to assess the systematic collection of adverse events in parenting intervention studies, providing specific recommendations and considerations for future studies [6]. Taher et. al. also provide insight via a case study of the steps required by regulators for the safety assessment of a digital mental health intervention [7]. They provide methods, scales and tools that can be adapted for use in other similar research areas. These two articles provide practical examples demonstrating clear steps for those in similar settings to apply.

Improving *trial efficiencies* has become an important consideration as trials have become more resource intensive and expensive to conduct. Trial staff (trial managers, monitors etc.) and clinical research staff have many competing demands on their time. The article by Black et. al. describes a new data system solution they have developed to facilitate timely and more efficient reporting of serious adverse events to the trial sponsor [8]. They offer insights suitable for UK clinical trial units to consider, but widespread availability of this solution is needed to enable improved efficiency nationwide.

The recent ‘*Guidance for best practices for Clinical Trials*’ from the World Health Organisation (WHO) highlights that there is “*a pressing need to promote and advance efficient and sustained well-designed and well-implemented clinical trials that address local health needs across all stages of clinical research in LMICs* [Iow and middle income countries] *and other resource-limited settings*” [9, 10]. Ayi-Ashong Bruce et. al. examine this issue in their article, highlighting the challenges faced by the Medical Research Council Unit in The Gambia when delivering vaccine trials [11]. They highlight the differences in health seeking behaviours, health systems and relevant sociocultural considerations that impact reporting of adverse events in such settings and share the strategies they have employed to overcome these challenges and how these can be adopted in other low-income settings.

Reducing *inequalities and maximising inclusion in research* is a recent priority for the WHO and National Institute for Health and Care Research (NIHR). This is now included in their strategies and guidance [9, 12]. In their commentary Hill et. al. discuss the challenges associated with the collection of safety data in neonatal clinical trials, especially in low and middle income countries where burden is often greatest [13]. This is a population highlighted by the WHO as being underserved by clinical trials [9]. The authors offer potential solutions to enable improved and proportionate safety data collection and reporting for future neonatal-focused trials. Li et. al. consider the impact of inequality and the consequence for the assessment of harm [14]. They raise that intervention harm often varies across populations due to biological and social factors, and there is a potential for interventions to exacerbate disparities. They stress the need to integrate equity considerations into research practices and trial methodologies through study design and practices such as inclusive participant recruitment.

Skommer et. al. offer a cautionary note for phase I trials where they raise the idea that while much focus is given to the physical health of participants in phase I trials, little consideration is given to their psychological wellbeing [15]. The authors argue that an individual’s personality traits will impact the frequency and intensity of adverse event reporting and further research is needed to understand the psychological characteristics of those participating in trials. While this is discussed for early phase trials there are aspects of their argument that could extend to later phase trials.

### Analysis

The *analysis* of harm outcomes in randomised controlled trials has been historically neglected and accepted good analysis practice is often disregarded [3]. There is an understanding within the clinical trials community that how we analyse harm outcomes in trials needs to change, and guidance on best practice across all types of harm data is needed. One challenging aspect is the population who should be included in the harm analysis, as the intervention effect may be an underestimate when participants are lost to follow-up or do not take the intervention. One solution to this is to use time-to-event analysis. Rufibach et. al. summarise the key findings from the SAVVY (Survival analysis for AdVerse events with VarYing follow-up times) project that examined different analytical approaches to account for varying follow-up times and competing events [16]. The authors provide practical recommendations to support implementation of this analysis.

### Reporting

CONSORT Harms 2022 provides guidance for *reporting* harms in RCT publications [17]. This recent update to the 2004 paper demonstrates how to integrate the checklist into the main CONSORT checklist [18]. What is still lacking is reporting that will support the readers to weigh up the harm with the ‘effectiveness’ results in a trial. In this series Totton et. al. suggest that benefit-risk methods are one tool that could be employed to present a more balanced report [19]. They use case-studies to demonstrate how adverse events can be presented alongside effectiveness outcomes and illustrate the impact on inferences.

A clinical study report (CSR) is a comprehensive report of a clinical trial’s results that are typically submitted to regulators as part of the drug approval process. The results within a CSR extend beyond the results presented in the journal article. Aronson et. al. introduce these and the contribution that they could play to enrich understanding of the benefit-risk profile for pharmacological interventions [20, 21]. In part one they provide a brief overview of the history of CSRs, noting the requirements from regulators (as covered by the International Council for Harmonisation (ICH) E3 guideline) [20, 22]. This sets the scene for part 2, a review of articles that integrated data from CSRs to assess the benefit-risk profile of interventions [21]. They find that “*when data from CSRs were taken into account the apparent benefits were less impressive and the adverse events more frequent*.” The authors conclude this has important implications for systematic reviews of interventions, but this seems as important for trialists and practitioners who use individual trial results to inform practice.

### Public involvement

This special series advertised research pertaining to adverse events, but in practice there are multiple terms used such as harm, safety and risk. Even within this editorial we have used each of these terms. While researchers in this field often still debate the most appropriate terminology, Phillips et. al. sought *public partners perspectives* on these terms in two replicated focus groups [23]. They found while researchers tend to debate the most appropriate term, the public actually think the most appropriate term depends on the context, including how serious the ‘event’ is. This could contribute to the ongoing debate amongst researchers. Authors also explored opinions on how harm gets communicated and they provided some key areas to improve public facing materials. This final article is an example of how public involvement can help us shape the way we undertake trial research.

## Summary

The aim of this special series was to bring together current research on best practice for collecting, analysing and presenting complex adverse event data in clinical trials. It provides an introduction into all aspects of methodology research needed to improve the way we assess intervention harm in trials. The articles look at early to late phase trials, simple to complex interventions, and consider different trial contexts, research inclusion and public opinion. They provide examples and recommendations that support much needed practical implementation advice for trialist. Collectively the articles indicate the scope of research needed for robust harm evaluation and inference, and how the whole pathway from collection to final inference needs careful attention to do this well. There remain many gaps for future research.

## References

[1] Deliver complex and innovative trials in the UK. Available from: https://www.nihr.ac.uk/support-and-services/industry/spotlights/complex-innovative-trials.

[2] Beckman RA, Natanegara F, Singh P, Cooner F, Antonijevic Z, Liu Y, et al. Advancing innovative clinical trials to efficiently deliver medicines to patients. Nat Rev Drug Discov. 2022;21(8):543–4. 35760887 10.1038/d41573-022-00109-y PMC9834420

[3] Cornelius VR, Phillips R. Improving the analysis of adverse event data in randomized controlled trials. J Clin Epidemiol. 2022;144:185–92. 34954021 10.1016/j.jclinepi.2021.12.023

[4] Briefing notes for researchers - public involvement in NHS, health and social care research. 2021. Available from: https://www.nihr.ac.uk/briefing-notes-researchers-public-involvement-nhs-health-and-social-care-research#tab-256881. Updated May 2024.

[5] Papaioannou D, Hamer-Kiwacz S, Mooney C, Sprange K, Cooper C, O’Cathain A. Recommendations on recording harms in randomised controlled trials of behaviour change interventions. BMJ. 2024;387:e077418. 39357901 10.1136/bmj-2023-077418 PMC11445694

[6] Frantz I, Foran HM, Lachman JM, Gardner F, McMahon RJ, Ogden T, et al. Adverse event assessment in a parenting programme: experiences from a multisite randomised controlled trial. Trials. 2024;25(1):547. 39154169 10.1186/s13063-024-08357-6 PMC11330034

[7] Taher R, Hall CL, Bergin ADG, Gupta N, Heaysman C, Jacobsen P, et al. Developing a process for assessing the safety of a digital mental health intervention and gaining regulatory approval: a case study and academic’s guide. Trials. 2024;25(1):604. 39252100 10.1186/s13063-024-08421-1 PMC11385814

[8] Black J, Julier P, Eldridge L, Barber VS. Streamlining electronic reporting of serious adverse events (SAEs) using the REDCap data collection system: the eSAE project. Trials. 2024;25(1):503. 39044237 10.1186/s13063-024-08317-0 PMC11267891

[9] WHO Team: Research for Health (RFH). Guidance for best practices for clinical trials. Geneva: World Health Organization; 2024. p. 76. ISBN: 978-92-4-009771-1. https://www.who.int/publications/i/item/9789240097711.

[10] Park JJH, Grais RF, Taljaard M, Nakimuli-Mpungu E, Jehan F, Nachega JB, et al. Urgently seeking efficiency and sustainability of clinical trials in global health. Lancet Glob Health. 2021;9(5):e681–90. 33865473 10.1016/S2214-109X(20)30539-8 PMC8424133

[11] Bruce AAA, Umesi AO, Bashorun A, Ochoge M, Yisa M, Obayemi-Ajiboye D, et al. Collecting and reporting adverse events in low-income settings—perspectives from vaccine trials in the Gambia. Trials. 2024;25(1):579. 39223604 10.1186/s13063-024-08419-9 PMC11370134

[12] Research inclusion strategy 2022-2027 2022. Available from: https://www.nihr.ac.uk/about-us/who-we-are/research-inclusion/strategy-2022-27. Updated March 2023.

[13] Hill LF, Obiero CW, Bekker A, Walker AS, Bielicki JA, Sharland M, et al. Safety reporting in neonatal clinical trials: reflections towards optimal, globally relevant approaches. Trials. 2025;26(1):46. 39924475 10.1186/s13063-025-08723-y PMC11808976

[14] Li T, Mayo-Wilson E, Shaughnessy D, Qureshi R. Studying harms of interventions with an equity lens in randomized trials. Trials. 2024;25(1):403. 38902776 10.1186/s13063-024-08239-x PMC11191320

[15] Skommer J, Gunesh K, Polasek TM. Personality vulnerabilities and adverse event reporting in phase 1 clinical studies. Trials. 2024;25(1):488. 39026376 10.1186/s13063-024-08321-4 PMC11256560

[16] Rufibach K, Beyersmann J, Friede T, Schmoor C, Stegherr R. Survival analysis for AdVerse events with VarYing follow-up times (SAVVY): summary of findings and assessment of existing guidelines. Trials. 2024;25(1):353. 38822392 10.1186/s13063-024-08186-7 PMC11143657

[17] Junqueira DR, Zorzela L, Golder S, Loke Y, Gagnier JJ, Julious SA, et al. CONSORT Harms 2022 statement, explanation, and elaboration: updated guideline for the reporting of harms in randomised trials. BMJ. 2023;381:e073725. 37094878 10.1136/bmj-2022-073725

[18] Ioannidis JA, Evans SW, Gøtzsche PC, et al. Better reporting of harms in randomized trials: an extension of the consort statement. Ann Intern Med. 2004;141(10):781–8. 15545678 10.7326/0003-4819-141-10-200411160-00009

[19] Totton N, Waddingham E, Owen R, Julious S, Hughes D, Cook J. A proposal for using benefit-risk methods to improve the prominence of adverse event results when reporting trials. Trials. 2024;25(1):409. 38909232 10.1186/s13063-024-08228-0 PMC11193225

[20] Aronson JK, Onakpoya IJ. Clinical study reports—a systematic review with thematic synthesis: part 1. History, contents and structure, definitions, and terminology. Trials. 2025;26(1):141. 40301986 10.1186/s13063-024-08710-9 PMC12042613

[21] Aronson JK, Onakpoya IJ. Clinical study reports—a systematic review with thematic synthesis: part 2. Studying benefits, harms, and the benefit to harm balance of pharmacological interventions. Trials. 2025;26(1):145. 40312342 10.1186/s13063-024-08671-z PMC12044985

[22] ICH topic E3 structure and content of clinical study reports. 1996. https://database.ich.org/sites/default/files/E3_Guideline.pdf. https://www.ema.europa.eu/en/ich-e3-structure-content-clinical-study-reports-scientific-guideline.

[23] Phillips R, Bi D, Goulão B, Miller M, El-Askary M, Fagbemi O, et al. Public perspective on potential treatment intervention harm in clinical trials—terminology and communication. Trials. 2024;25(1):573. 39215336 10.1186/s13063-024-08418-w PMC11365119

